# A high-power, high-repetition-rate THz source for pump–probe experiments at Linac Coherent Light Source II

**DOI:** 10.1107/S1600577520005147

**Published:** 2020-06-15

**Authors:** Z. Zhang, A. S. Fisher, M. C. Hoffmann, B. Jacobson, P. S. Kirchmann, W.-S. Lee, A. Lindenberg, A. Marinelli, E. Nanni, R. Schoenlein, M. Qian, S. Sasaki, J. Xu, Z. Huang

**Affiliations:** a SLAC National Accelerator Laboratory, Menlo Park, CA 94025, USA; b Argonne National Laboratory, Lemont, IL 60439, USA

**Keywords:** THz, XFEL, pump–probe, femtosecond, synchronization

## Abstract

Experiments using a THz pump and an X-ray probe at an X-ray free-electron laser (XFEL) facility like the Linac Coherent Light Source II (LCLS II) require frequency-tunable (3 to 20 THz), narrow bandwidth (∼10%), carrier-envelope-phase-stable THz pulses that produce high fields (>1 MV cm^−1^) at the repetition rate of the X-rays and are well synchronized with them. In this paper, a two-bunch scheme to generate THz radiation at LCLS II is studied: the first bunch produces THz radiation in an electromagnet wiggler immediately following the LCLS II undulator that produces X-rays from the second bunch. The initial time delay between the two bunches is optimized to compensate for the path difference in THz transport. The two-bunch beam dynamics, the THz wiggler and radiation are described, as well as the transport system bringing the THz pulses from the wiggler to the experimental hall.

## Abstract 2

A two-bunch scheme to generate high-power, high-repetition-rate THz radiation for pump–probe experiments at LCLS II is studied. The two-bunch beam dynamics, the THz wiggler and radiation are described, as well as the transport system bringing the THz pulses from the wiggler to the X-ray experimental hall.

## 1. Introduction   

The effective coupling of advanced sources of terahertz (THz) radiation with X-ray free-electron laser (XFEL) capabilities will open many new science opportunities. THz oriented workshops at SLAC (Gaffney *et al.*, 2012 ▸), ANL (Wen *et al.*, 2013 ▸), FERMI (Perucchi *et al.*, 2013 ▸), Eu-XFEL (Zalden *et al.*, 2018 ▸) and elsewhere in the past eight years have highlighted the demand for the capability to carry out these types of experiments. At the same time, significant advances have been made in the development of advanced table-top THz capabilities, and many of these capabilities have been applied to time-resolved THz/X-ray experiments at various XFEL facilities (Kubacka *et al.*, 2014 ▸; Chen *et al.*, 2016 ▸; Gray *et al.*, 2018 ▸; Hoffmann *et al.*, 2018 ▸; Kozina *et al.*, 2019 ▸).

The new science opportunities presented by a high-repetition-rate FEL such as LCLS II create new opportunities, demands, and challenges for THz sources that go beyond what has been considered in previous workshops. Some critical capability gaps can already be identified that appear to be beyond the projected properties of table-top sources. Among them are intense sources in the well known THz gap between 3 and 20 THz. For field-driven effects, a broadband, single-cycle THz pulse with a peak electric field strength of 10 MV cm^−1^ can approach the atomic bonding strength in matter. For resonant excitation, a tunable, narrow bandwidth (∼10%) source with an energy of at least 10 µJ pulse^−1^ is desired. The THz requirements summarized in Table 1 ▸ are the target parameters for the proposed THz source described in this paper.

Although a dedicated accelerator with a beam energy of tens of MeV can achieve some of these capabilities [see Green *et al.*, 2016 ▸) as well as a detailed study conducted a few years ago at SLAC (Schmerge *et al.*, 2015 ▸)], it becomes increasingly difficult to reach 10 THz and above with significant pulse energy using a comparatively low-energy electron beam. In addition, a high-repetition-rate stand-alone accelerator becomes rather complex after considering the technical issues of synchronization with the X-rays, machine protection, shielding, and THz transport through the shielding. Intense, high-frequency THz pulses from SLAC high-energy, strongly compressed beams (LCLS and FACET) have been generated in the past using coherent transition radiation or edge radiation (Wu *et al.*, 2013 ▸), but they are limited to single-cycle and cannot be used for narrow-bandwidth THz generation. Inspired by the pioneering work of the FLASH THz beamline (Tiedtke *et al.*, 2009 ▸), we propose to install an electromagnet (EM) wiggler after the LCLS II soft X-ray (SXR) undulators and to use a two-bunch scheme to produce intense THz pulses for high-repetition-rate pump–probe experiments. The EM wiggler allows generation of more than 100 µJ narrow-bandwidth (10%) high-frequency THz radiation, and also allows broadband THz radiation of similar pulse energy by controlling the number of wiggler periods independently. We note that an earlier study of a THz source for Eu-XFEL has a similar two-bunch scheme while employing a high-field superconducting wiggler as it operates at a much higher electron energy than LCLS II (Tanikawa *et al.*, 2019 ▸).

This paper is organized as follows. We start by reviewing the state-of-art laser-based THz sources. Then we describe the proposed accelerator approach with the two-bunch scheme and the associated beam dynamics studies. Following the two-bunch section, we discuss the THz wiggler design, the radiation characteristics, as well as the optical transport system for the LCLS II. The paper concludes with a brief summary.

## 2. Review of laser-based THz sources   

Laser-based THz generation has been successfully employed for user experiments at XFEL facilities such as LCLS (Kubacka *et al.*, 2014 ▸; Chen *et al.*, 2016 ▸; Gray *et al.*, 2018 ▸; Hoffmann *et al.*, 2018 ▸; Kozina *et al.*, 2019 ▸). Here, Ti:sapphire-based femtosecond laser systems at 800 nm are used for THz generation using various nonlinear optical methods (Hoffmann & Fülöp, 2011 ▸). This eliminates the need for THz beam transport over long distances and enables the choice of THz properties based on the requirements of the particular user experiments. Because LCLS I runs at a 120 Hz repetition rate, it is possible to use relatively high pulse energies of up to 20 mJ at 800 nm to enable high nonlinear conversion efficiency and thus high THz pulse energies and peak THz fields. A very efficient way to generate single-cycle THz pulses with the 800 nm femtosecond pulse directly is the use of optical rectification in LiNbO_3_ using the tilted pulse front method (Hebling *et al.*, 2002 ▸). Other options include optical rectification in organic nonlinear crystals (Zhang *et al.*, 1992 ▸; Hauri *et al.*, 2011 ▸), or difference frequency generation in gallium selenide for shorter wavelength THz and mid-IR pulses. In this case an additional optical parametric amplifier (OPA) stage is needed to convert the 800 nm femtosecond laser pulse to longer wavelengths before it can be used for THz generation.

For LCLS II we are currently developing a novel optical parametric chirped amplifier (OPCPA) system that will be able to operate at 100 kHz and provide up to 1 mJ pulse energy with sub-20 fs pulse duration at 800 nm (Mecseki *et al.*, 2019 ▸). This laser system will be synchronized to the X-ray pulses from the FEL and used for pump–probe experiments in the LCLS Near Experimental Hall. Further upgrades include a tunable near-infrared OPCPA for wavelengths in the range 1500–1800 nm (Windeler *et al.*, 2019 ▸). The main challenge for laser-based THz generation methods for LCLS II is the comparatively low pulse energy – limiting generation efficiency – and control of thermal issues due to the high average power when using the full 100 kHz rate. We have recently carried out experiments to assess the scaling of THz generation to 100 kHz repetition rate using this laser system. We found that optical rectification in LiNbO_3_ scales well with high average powers due to favorable thermal properties of the material. The high spectral bandwidth of the OPCPA output at 800 nm does not favor tilted pulse front generation with a grating, but an echelon can be used instead (Ofori-Okai *et al.*, 2016 ▸) and THz pulse energies on the order of 100 nJ can be achieved. Alternatively, it is possible to spectrally broaden and recompress the picosecond pump laser of the OPCPA system. Laser pulses of 5 mJ at ∼1064 nm and 100 fs duration can be achieved. When these are used for THz generation in LiNbO_3_ with the tilted pulse front method, we were able to obtain 150 mW of THz average power corresponding to about 1.5 µJ THz pulse energy. When organic nonlinear crystals are used for THz generation at 1500 nm pump wavelength, the high average power becomes problematic. Thermal properties of these crystals are not favorable and damage becomes an issue at high repetition rates when an average power of 1 W cm^−2^ is exceeded. However this method may be successfully used for LCLS II experiment that do not require the full 100 kHz repetition rate.

## 3. Two-bunch scheme   

**Figure 1:**
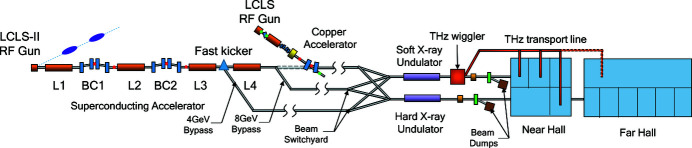
LCLS II layout showing the linac, fast kicker, soft and hard X-ray undulators, the proposed THz wiggler and transport line, and the Near and Far Experimental Halls.

In this section, we propose a two-bunch scheme for the LCLS II (see Fig. 1 ▸), which can deliver a burst of intense THz radiation and an X-ray pulse with the adjustable time separation needed for pump–probe experiments. Two electron bunches with a suitable time delay are generated at the gun and sent through the linac (Decker *et al.*, 2015 ▸; Penco *et al.*, 2018 ▸). After acceleration to ∼4 GeV, the beams are extracted and sent to the SXR line, where the XFEL undulator is followed by a ten-period wiggler that produces intense THz radiation. Since the THz pump must arrive earlier than the X-ray probe, the first bunch (referred as the THz bunch) is used to produce a THz pulse while the second one (the FEL bunch) lases in the FEL undulator. The initial time delay of the two beams compensates for the difference in the longer, less direct, transport path of the THz to the user station, compared with the nearly straight path of the X-rays. When the time delay between the two beams is a multiple of the 5.4 ns radiofrequency (RF) period in the gun, we can generate two identical electron bunches. However, the nominal LCLS II electron bunch, with a length of ∼100 fs and a charge of 100 pC, is not optimal for producing THz radiation at the high end of our frequency range (>10 THz). A higher charge and a shorter bunch length are always desirable for THz generation. The generation and compression of the THz bunch needs to be adjusted accordingly, but without altering the baseline settings of the LCLS II beamline designed to deliver electron bunches with beam dynamics optimized for the XFEL. So in practice the adjustable beamline parameters for the THz bunch are mostly limited to those of the photocathode laser, including its pulse energy (to produce more charge), length, transverse spot size, and injection phase at the gun.

**Figure 2:**
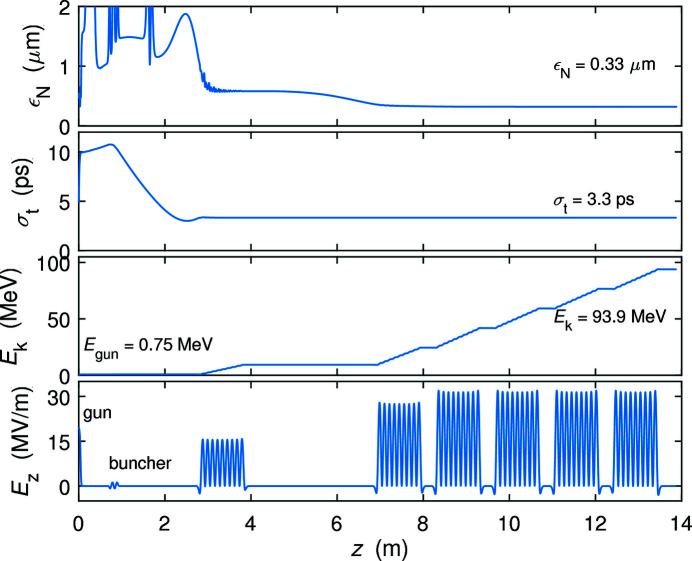
The baseline design of the LCLS II injector for 100 pC, including the normalized emittance (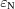), r.m.s. bunch length (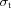), beam kinetic energy (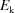) and longitudinal acceleration field (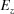).

The LCLS II injector includes a normal-conducting continuous-wave (CW) RF gun operating at 186 MHz (7th sub-harmonic of 1.3 GHz), a 1.3 GHz buncher and superconducting accelerator (Schmerge *et al.*, 2014 ▸). The injector can simultaneously deliver bunches at a high repetition rate of up to 1 MHz and with a normalized emittance of <0.6 (0.4) µm at 300 (100) pC per bunch and peak current >30 (12) A. Following the buncher, a single standard TESLA-style cryomodule with eight nine-cell SRF cavities accelerates the beam from <1 to approximately 100 MeV. Fig. 2 ▸ shows the baseline design of the LCLS II injector for a 100 pC bunch charge simulated by ASTRA (Floettmann, 2017 ▸), including the normalized emittance, root-mean-square (r.m.s.) bunch length, beam kinetic energy and longitudinal acceleration field. The detailed parameters of the simulation are presented in Table 2 ▸. The drive laser has a 32.9 ps flat-top profile and the injection phase in the gun is about −6.6° (relative to the phase with maximum acceleration in each cavity, defined as 0°). The phase of the buncher is set to −80.3° for beam compression. The beam charge of the THz bunch can be varied by controlling the laser pulse energy. The power-handling capability of the LCLS II beam dump allows the delivery of both a 100 pC FEL bunch and an additional THz bunch with a bunch charge of up to 200 pC at ∼100 kHz.

### 3.1. Velocity bunching of the THz bunch   

**Figure 3:**
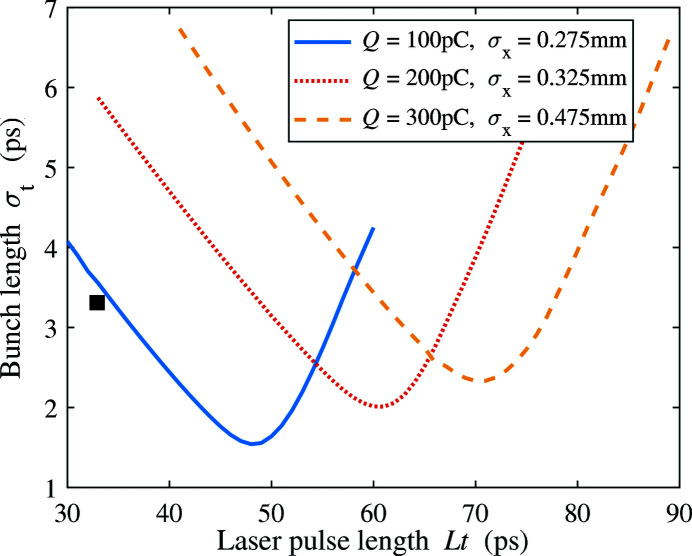
The r.m.s. bunch length at the exit of the injector for different laser pulse lengths. The bunch charge and laser spot size are fixed for each scan. The black square represents the parameters of the nominal 100 pC LCLS II beam.

**Figure 4:**
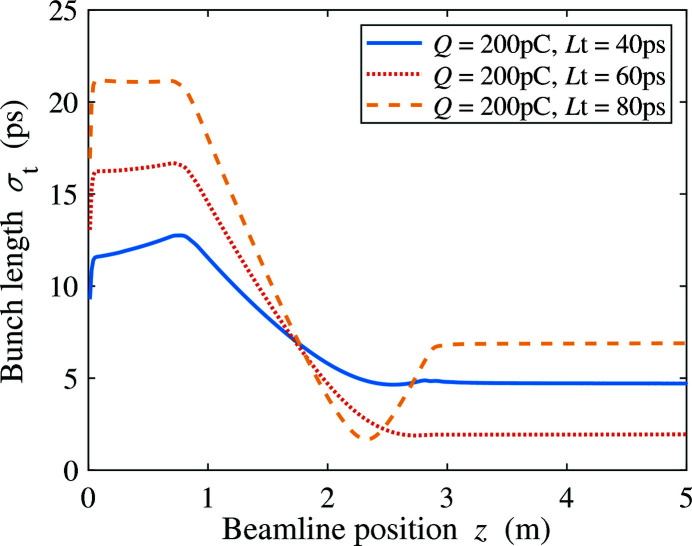
Bunch length evolution of a 200 pC beam along the injector beamline with laser pulse length 40, 60 and 80 ps.

The compression of the THz bunch can be achieved by the combination of velocity bunching in the injector and magnetic compression in the two downstream compressor chicanes. Since the RF wavelength of the gun is much longer than the laser pulse length, velocity bunching in the gun can be ignored. The beam expands gradually due to its space charge before it enters the buncher. There, acceleration far off-crest causes an energy chirp that compresses the electron bunch in the following drift. Its length is then frozen once the beam is accelerated to high energy, as shown in Fig. 2 ▸. Fig. 3 ▸ shows the r.m.s. bunch length of the electron beam at the exit of the injector under different initial laser pulse lengths for bunch charges of 100, 200 and 300 pC. The laser spot size on the cathode is increased to support higher charge. For each beam charge, there is an optimal laser pulse length that minimizes the bunch length after the injector, which can be explained by the space charge effect. We present the bunch length evolution of the 200 pC beam with initial laser pulse length 40, 60 and 80 ps in Fig. 4 ▸. Since the beam energy is very low (<1 MeV) before the buncher, a short laser pulse creates an energy chirp opposite to that induced by the buncher, reducing the compression factor. The optimal value of the laser pulse length corresponds to the full compression of velocity bunching. A long laser pulse leads to the over-compression of the beam and longer bunch length.

**Figure 5:**
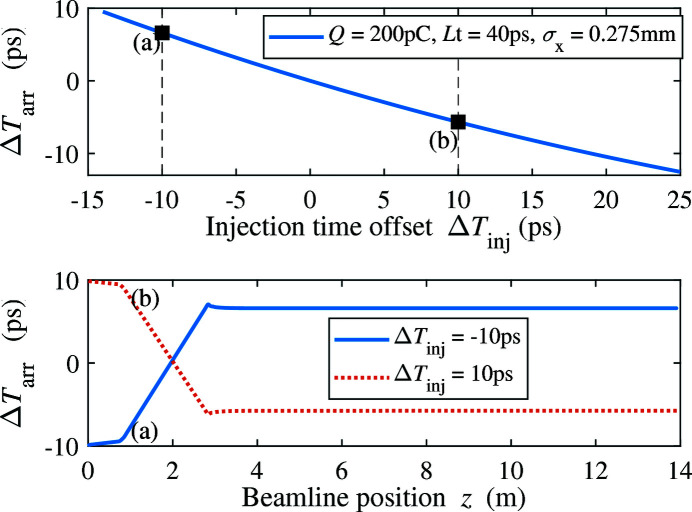
Top: beam arrival time of 200 pC THz bunch after the injector for different injection time offsets. Bottom: beam arrival time evolution along the beamline when the injections time offset is −10 and 10 ps.

**Figure 6:**
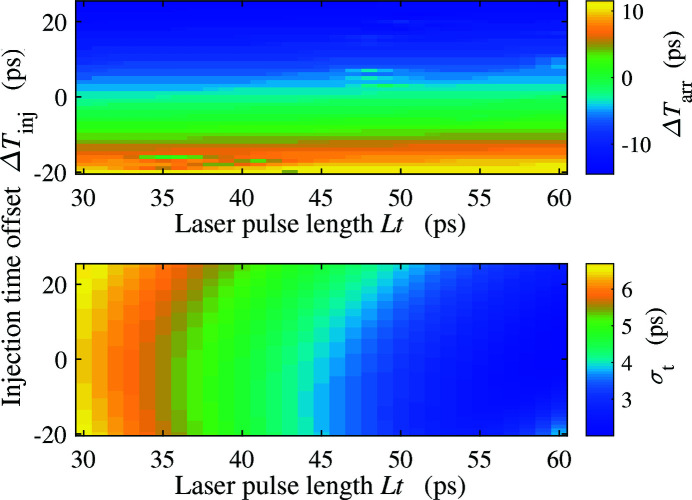
Beam arrival time (top) and r.m.s. bunch length (bottom) of the 200 pC THz bunch for different laser pulse length and injection time offset.

In addition to the bunch length, the arrival time of the electron beam at the exit of the injector, which may change the downstream compression, is an important parameter for THz bunch control and THz generation as well. The beam arrival time depends on the injection time of the laser in the gun. Taking the 200 pC THz bunch as an example, we vary the injection time in the gun and compare the arrival time with that of the nominal 100 pC FEL bunch, which is shown in Fig. 5 ▸. There is a clear linear dependence between the beam arrival time and the injection time offset. The negative correlation indicates that the electron with an early injection time (negative offset) needs more time to pass through the beamline, leading to a late arrival time (positive offset). This negative correlation is due to the strong dependence of beam drift velocity on the beam energy when the beam energy is small. In Fig. 5 ▸ we also present the beam arrival time offset at a given position along the beamline to the nominal FEL bunch under different initial injection time offsets (−10 and 10 ps). Fig. 6 ▸ shows the beam arrival time and r.m.s. bunch length of the 200 pC THz bunch for different laser pulse length and injection time offset. It is obviously observed that in the injector we can control the beam arrival time and r.m.s. bunch length independently by the injection time offset and laser pulse length, respectively.

### 3.2. Two-stage magnetic compression of THz bunch   

After the injector, the electron beam is accelerated in three 1.3 GHz linac sections (L1, L2, L3), with a 3.9 GHz harmonic cavity (L1C) as a linearizer in L1. The three sections are separated by two magnetic bunch-compressor chicanes (BC1, BC2). The tunable parameter for the compression control of the THz bunch is the arrival time at the first linac (L1). The acceleration phases of the downstream linacs and the modified *R*_56_ of the two chicanes will be updated accordingly. With the output of the injector, *LiTrack* (Bane & Emma, 2005 ▸) is used to simulate the two-stage acceleration and compression. *LiTrack* is a 1D simulation code, which tracks only the longitudinal phase space but neglects transverse beam dynamics and collective effects other than longitudinal wakefields. The baseline parameters of the two-stage acceleration and compression are presented in Table 3 ▸.

**Figure 7:**
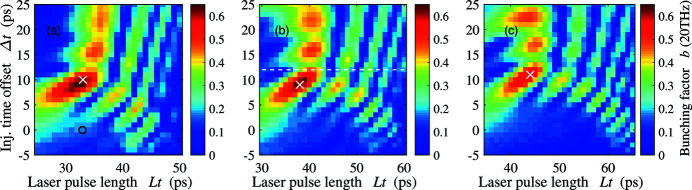
Bunching factor at 20 THz of the THz bunch at the entrance of the undulator for different laser pulse length and injection time offset. The beam charge is 100 pC (*a*), 200 pC (*b*) and 300 pC (*c*). The black circle represents the nominal 100 pC FEL bunch and the white crosses represent the optimal points for each beam charge, whose longitudinal phase space is shown in Fig. 8 ▸. The bunching factors along the dashed line in (*b*) at 12 ps injection time offset are presented in Fig. 9 ▸.

**Figure 8:**
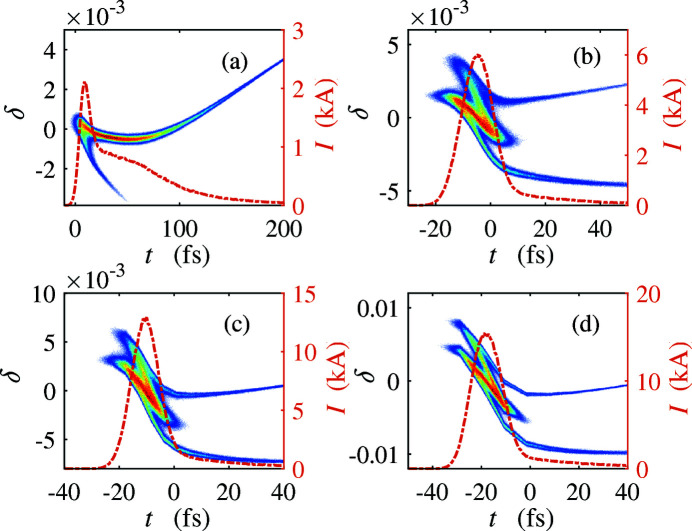
Longitudinal phase space of the FEL bunch (*a*) and optimal THz bunches with beam charge 100 pC (*b*), 200 pC (*c*) and 300 pC (*d*).

**Figure 9:**
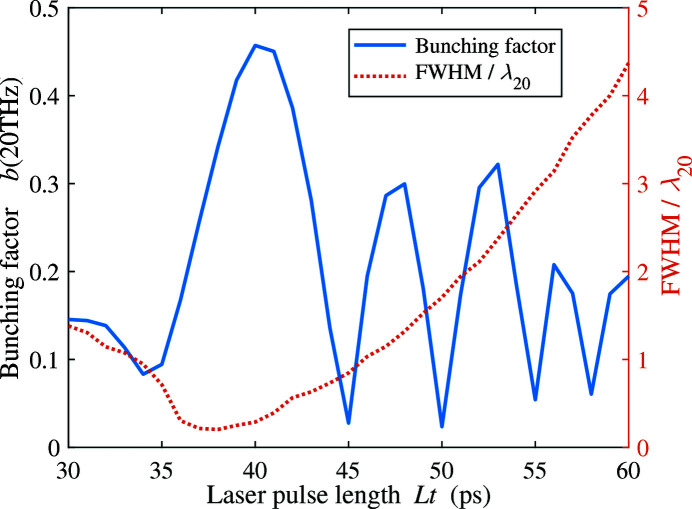
Bunching factor and FWHM ofthe 200 pC THz bunch for different laser pulse length at 12 ps injection time offset [dashed line in Fig. 7(*b*) ▸]. The FWHM is normalized by the radiation wavelength of 20 THz.

In Fig. 7 ▸ we present the bunching factor at 20 THz of the THz bunch at the entrance of the undulator for different laser pulse lengths and injection time offsets. The optimizations are performed for three beam charges. The bunching factor here is defined as
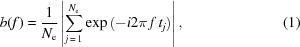
where *f* is the bunching frequency, *N*_e_ is the number of electrons and *t*_*j*_ is the arrival time of the *j*th electron at the undulator. For the 100 pC bunch, the bunching factor under nominal beamline settings is only 0.18. However, with the same laser pulse length, when the laser pulse is injected earlier by ∼10 ps, the downstream compression of the beam increases the bunching factor to 0.63. With more charge, we find the optimal injection time offset is almost the same while the laser pulse length increases with the beam charge (38 ps for 200 pC and 44 ps for 300 pC). Fig. 8 ▸ shows the longitudinal phase space of the FEL bunch and the optimal THz bunches with different beam charge at the entrance of the undulator. Compared with the nominal FEL bunch, we can observe a shorter bunch length, a higher peak current, and a larger energy spread on the compressed THz bunches. In Fig. 9 ▸ we show the bunching factor and the FWHM of the 200 pC THz bunch versus the laser pulse length and at a fixed 12 ps injection time offset [dashed line in Fig. 7(*b*) ▸]. The FWHM is normalized by the radiation wavelength of 20 THz. Note that the bunching factor versus the laser pulse length has a clear periodic evolution. The bunching factor reaches local minimum where the FWHM is about a multiple of radiation wavelength at 20 THz, which accounts for the periodic pattern in Fig. 7 ▸.

**Figure 10:**
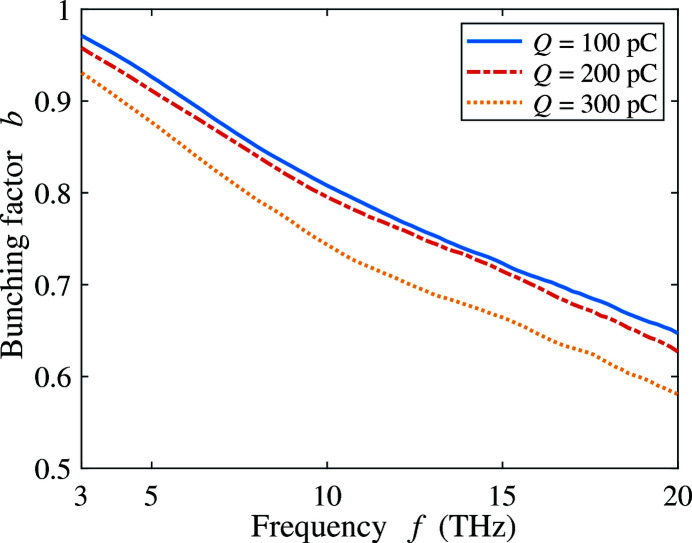
Optimal bunching factor at the frequency range from 3 to 20 THz with beam charge 100, 200 and 300 pC.

For the frequency range from 3 to 20 THz, similar optimization simulations provide the optimal bunching factor at different beam charge, as shown in Fig. 10 ▸. The optimal bunching factor of the 100 pC THz bunch has a linear dependence on the bunching frequency, which can be fitted as *b*(*f*) = 1 − 0.019*f*  [THz]. The optimal bunching factors of the 200 pC and 300 pC bunches are both very close to the one for the 100 pC bunch, which indicates that in the current beamline settings the bunching factor is not limited by the beam charge. Higher beam charge is always desirable even for the high-frequency THz generation in our design. The electron beam with high charge and high bunching factor can be a very powerful source in THz generation.

### 3.3. Beam operation mode   

The LCLS II high-energy upgrade will provide both 4 GeV and 8 GeV electrons, with the higher-energy beam traveling through a second 8 GeV bypass line (Fig. 1 ▸). Bunches at both energies can be sent to the SXR and HXR undulator lines. However, the proposed THz wiggler can still use 4 GeV as described here, which results in different beam operation modes for the pump–probe experiments: THz-SXR (4 GeV), THz-SXR (8 GeV), THz-HXR (4 GeV) and THz-HXR (8 GeV). Different operation modes put various requirements on the beam spreader system in the facility.

In the proposed two-bunch scheme, fast kickers are used to kick one or both of the two bunches. When we need to kick both bunches, for example in the mode of THz-SXR (4GeV), the flatness of the kicker voltage waveform after L3 must be longer than the time separation of the two bunches (∼100 ns). Presently the magnetic field waveform is a triangle with ∼120 ns rise/fall time (Beukers *et al.*, 2018 ▸). However, much effort has been devoted to extending the flatness of the kicker voltage waveform. Minor control modifications to the equipment are necessary to allow longer pulse operations. The drawback is that the allowed repetition rate scales inversely with pulse width, since the overall duty cycle is limited. For a 108 ns-wide flat-top, the repetition rate would be limited to around 200 kHz (Beukers, 2019 ▸).

When we need bunches at different energies — 4 GeV for THz and 8 GeV for the XFEL — the THz bunch is kicked from the linac to the low-energy bypass line after L3, but the XFEL bunch continues through L4 to gain more energy before entering the high-energy bypass. In this case, the rise and fall times of the kicker voltage waveform should be below the time separation of the two bunches, which is not included in the baseline design. The turn-on/turn-off time is ∼120 ns for a combined pulse width of ∼240 ns. However, it is possible to lower the kick amplitude to decrease the turn-on/turn-off time. Ongoing kicker R&D at LCLS II for SXR/HXR pump–probe experiments should satisfy this requirement as well.

## 4. THz wiggler design   

**Figure 11:**
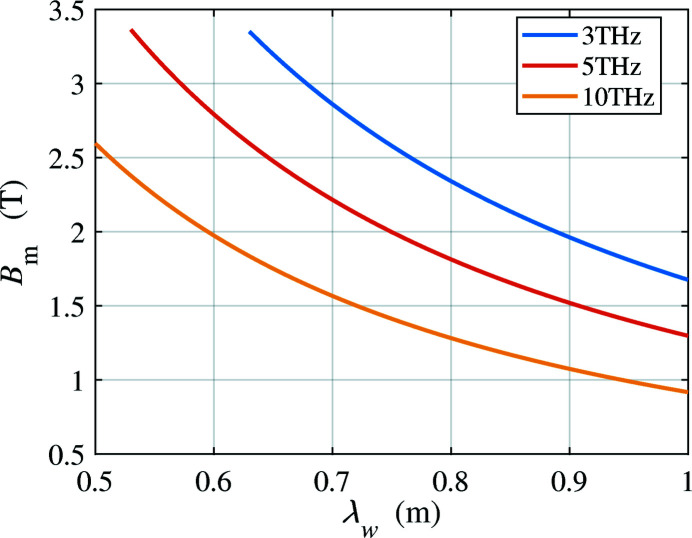
Magnetic field versus wiggler period when using a 4 GeV beam to generate 3, 5 and 10 THz radiation.

The parameters of the THz wiggler are optimized for a 4 GeV electron beam energy and radiation frequencies between 3 and 20 THz. We choose ten wiggler periods to produce THz radiation with a 10% bandwidth. The resonance condition can be expressed as
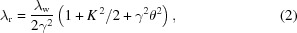
where 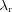 is the radiation wavelength, 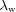 is the wiggler period, γ is the Lorentz factor and θ is the observation angle away from the longitudinal direction. *K* is the dimensionless undulator deflection parameter. A practical engineering formula for the deflection parameter is *K* ≃ 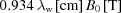 with maximum magnetic field 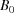. For fixed beam energy and undulator settings, the THz radiation on-axis has the highest frequency. The frequency gradually decreases as the emission angle theta to the axis increases. Fig. 11 ▸ shows the required peak magnetic field on-axis for different wiggler periods, for radiation at 3, 5 and 10 THz. The main limitation in practice is the available magnetic field with a wiggler gap wide enough to allow for diffraction at these long wavelengths (3 THz corresponds to 100 µm). It is preferable to avoid the cost and complexity of a superconducting wiggler, but fields in a room-temperature wiggler cannot practically exceed 2.5 T. We consider two wiggler technologies for THz generation, based on hybrid permanent magnets and electromagnets.

### 4.1. Hybrid permanent-magnet wiggler   

**Figure 12:**
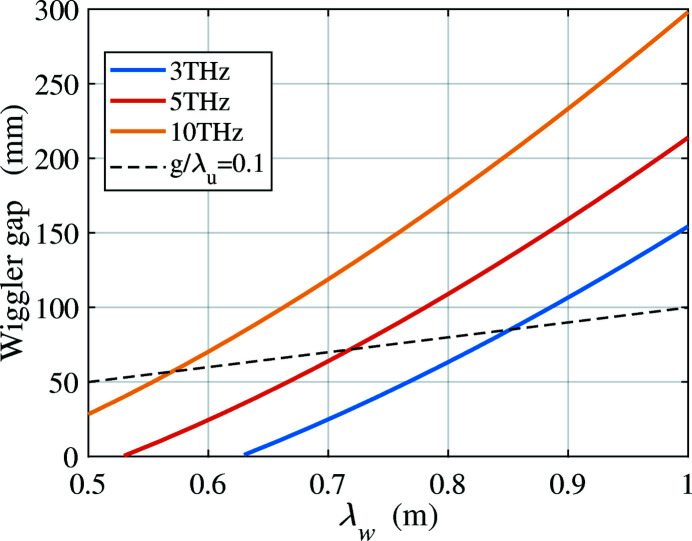
Gap in a hybrid permanent-magnet wiggler versus wiggler period when using 4 GeV beam to generate 3, 5 and 10 THz radiation.

A numerical fit to the peak field on-axis of a hybrid permanent-magnet wiggler, as a function of gap *g* and period 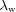, follows (Halbach, 1981 ▸)
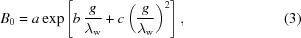
with 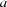 = 3.381, 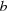 = −4.730, and 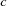 = 1.198. Based on this, the wiggler gap consistent with Fig. 11 ▸ is shown in Fig. 12 ▸. Note that the equation is usually valid over the range 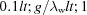. However, for a large wiggler period, 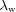 = 0.77 m, equation (3) is still applicable to the case of a 50 mm gap to achieve 2.5 T magnetic field, which is resonant at 3 THz with 4 GeV. Diffraction makes this long wavelength the most difficult case. For a fixed 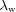, the lower wiggler parameter *K* needed for higher frequencies reduces the field and so widens the gap.

### 4.2. Electromagnet wiggler   

**Figure 13:**
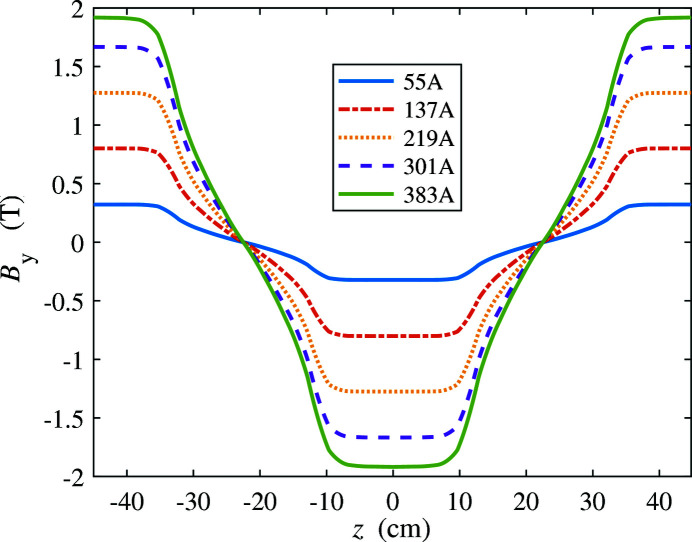
Field distributions along the *z*-axis over one 90 cm wiggler period at different current settings, for a gap of 50 mm.

In an electromagnet wiggler, the gap is fixed and the magnetic field is varied by changing the current in the coils. A conventional electromagnet can reach 2 T. In this case, we can produce 3 THz radiation with a wiggler period of 90 cm at 4 GeV. Field distributions along the *z* axis over one wiggler period at different current settings were simulated as shown in Fig. 13 ▸, for a gap of 50 mm.

**Figure 14:**
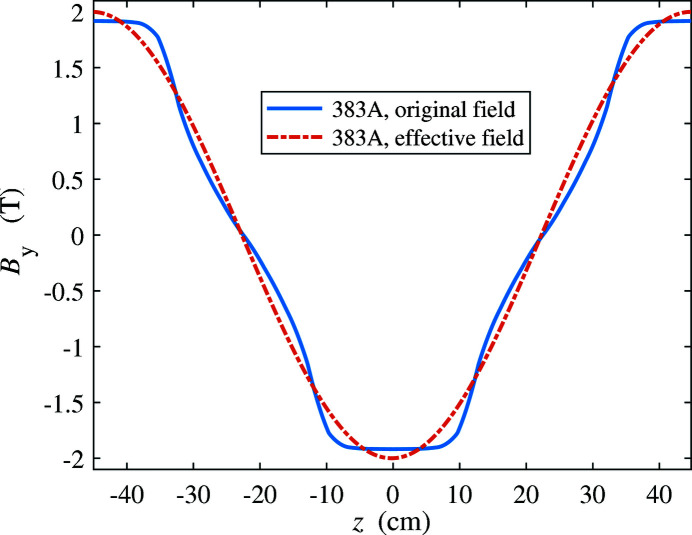
Effective field distribution for the 383 A case of Fig. 13 ▸.

The effective field was calculated by keeping the first term of the Fourier coefficient. Fig. 14 ▸ shows an example of the effective field for 383 A. The effective fields for other current settings, calculated in the same way, are shown in Table 4 ▸, with the corresponding wiggler *K* and radiation frequency.

**Figure 15:**
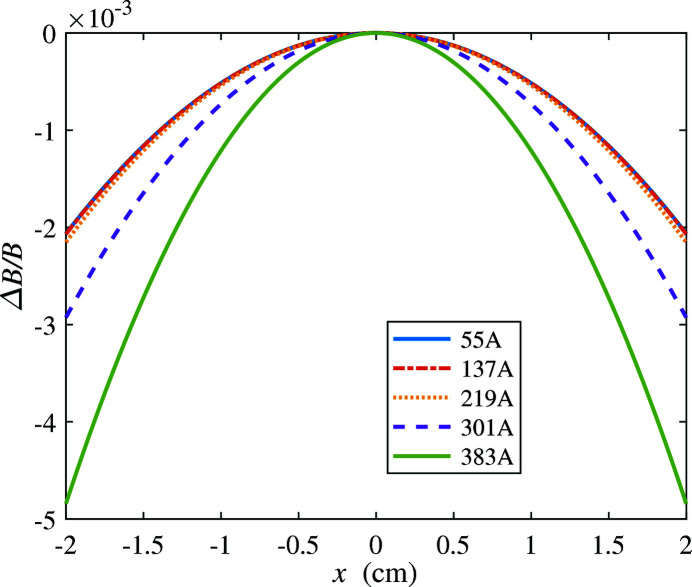
Roll-off of the wiggler field with horizontal offset from 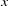 = 0 at the center of the pole.

Fig. 15 ▸ shows the simulated field roll-off in the horizontal (*x*) direction. For the highest field, 2 T, the field drops by 2% at an offset of 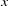 = ±2 cm. This is adequate as the wiggler amplitude at this field strength is about 2 mm.

The force between opposing poles is 73.6 kN, or 147 kN per period, for a current of 383 A. The temperature rise at a current of 383 A is approximately 12°C. The conductor cross-section is 10 mm × 10 mm with a 5 mm-diameter water hole; a water pressure of 0.62 MPa is assumed. Each pole is surrounded by one coil, each coil has six layers, and each layer has 20 turns. Each coil has three separate cooling-water loops. The power dissipated in each coil at 383 A is 5.2 kW.

An additional advantage of the electromagnet wiggler is that the magnetic field of each pole can be independently controlled with separate power supplies. For example, a single cycle of THz radiation can be generated by powering only one wiggler period while the supplies for the other nine periods remain off. The total radiated energy is about the same for a single-period wiggler as for a ten-period one, although the spectral energy density is ten times lower. Nevertheless, in the following discussions, we focus on the ten-period case for the illustration purpose.

In passing we note that the EM wiggler parameter *K* is very large and is easily controllable by the coil current. By reducing the wiggler parameter *K*, the resonant undulator frequency can cover the range from THz to visible and up to extreme ultraviolet (EUV). In fact the shortest wavelength reachable by such an EM wiggler at 4 GeV electron energy is around 7 nm. If the electron bunch can be shaped to have features on the order of the resonant wavelength (Tibai *et al.*, 2014 ▸), coherent visible or EUV radiation can be generated to provide versatile pump sources for pump–probe experiments at LCLS II.

## 5. THz radiation characteristics   

Intense THz radiation can be generated by sending the compressed beam to the ten-period wiggler which is located downstream of the SXR undulator line. The microbunching instability driven by the beam collective effects can degrade the beam quality of the THz bunch and subsequently suppress its lasing in the SXR undulator. In this section, we present the properties of the generated THz radiation, including the estimation of THz pulse energy and the calculation of the transverse distribution.

### 5.1. THz pulse energy   

The total achievable THz pulse energy depends on the specific layout of the wiggler and transport system (THz mirrors). Here we can estimate the pulse energy by the bunching factor spectra and the simplified equation about the single-electron radiation energy in the central cone defined as 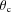 ≃ 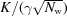 by Kim *et al.* (2017 ▸),
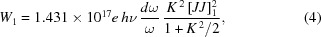
where 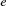 = 1.6 × 10^−19^ C is the elementary charge, 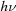 is the radiated photon energy and 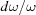 is the bandwidth of interest. The Bessel function factor
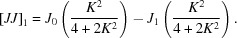
The total pulse energy can be written as (Saldin *et al.*, 2005 ▸)
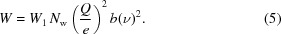
Here 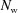 is the number of wiggler periods, *Q* is the beam charge and 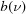 is the bunching factor at the radiation frequency ν.

**Figure 16:**
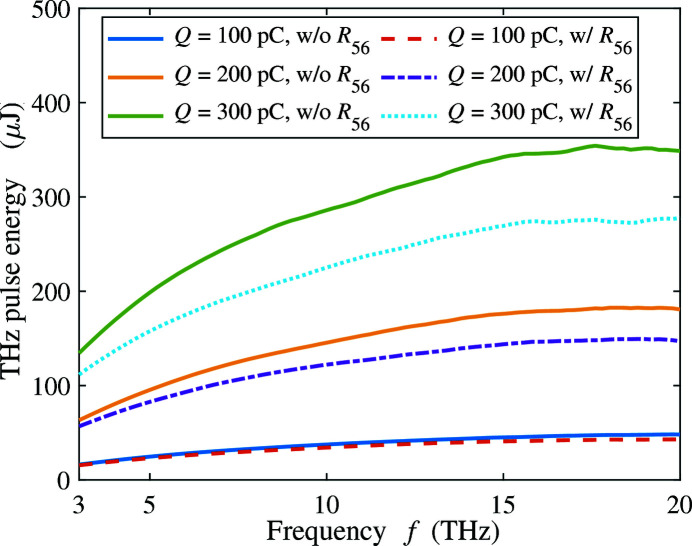
The estimated THz pulse energy of different beams versus the radiation frequency with and without the consideration of *R*_56_ of the wiggler.

Another effect that needs to be considered in radiation energy estimation is the compression or decompression effect due to the *R*_56_ of the wiggler (Geloni *et al.*, 2019 ▸). There is a large energy chirp on the beam where the energy of the beam head is higher than the tail, which is introduced by the longitudinal space charge force, the wakefield along the beamline and the short-range coherent synchrotron radiation (CSR) in the wiggler (MacArthur *et al.*, 2019 ▸). The *R*_56_ of each wiggler period will stretch the beam and reduce the bunching factor, especially in the high frequency range. In Fig. 16 ▸ we present the estimated pulse energy of different beam charge in the frequency range 3–20 THz with and without consideration of *R*_56_ of the wiggler. The CSR-induced energy loss and the rotation of phase space are calculated period by period. The THz energies are calculated based on the updated bunching factor of the beam after each period. In the current beamline settings of LCLS II, larger charge produces more pulse energy. The consideration of the wiggler *R*_56_ reduces the total pulse energy, especially for high beam charge. With 200 pC we can produce ∼120 µJ at 10 THz and ∼145 µJ at 20 THz. With 300 pC beam charge, more than 200 µJ pulse energy can be achieved over the frequency range from 5 to 20 THz.

### 5.2. Angular distribution of THz radiation   

**Figure 17:**
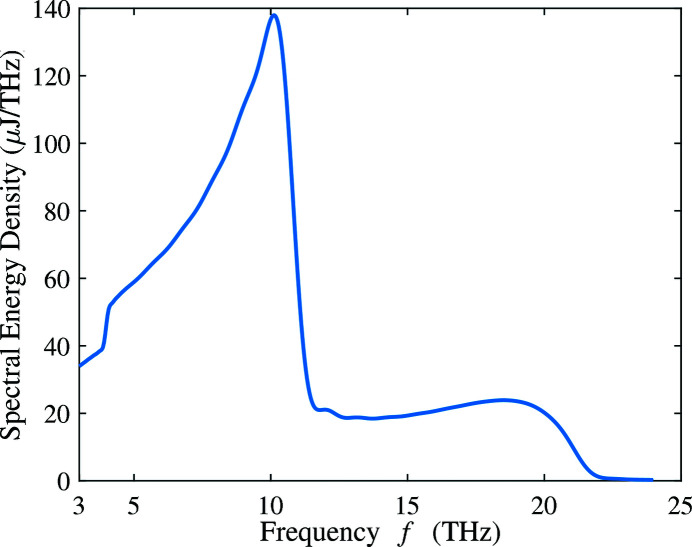
Spectrum of the THz radiation over the full bandwidth of the 200 pC THz bunch with the first and the second harmonics. The resonance frequency on-axis is set at 10.8 THz.

**Figure 18:**
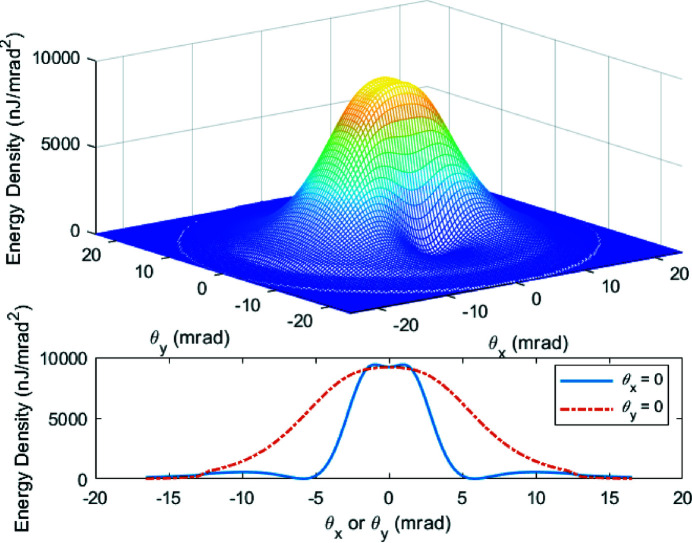
Far-field distribution of the THz radiation calculated by equation (6) over the full bandwidth of the 200 pC THz bunch with the first and the second harmonics. The resonance frequency on-axis is set at 10.8 THz.

In the THz wiggler, the dimensionless undulator deflection parameter 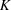 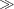 1 for which the maximum deflecting angle is larger than the natural radiation opening angle of about 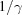. With the assumption that the maximum angle of the electron trajectory stays small relative to unity, 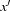 ≃ 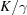 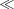 1, the far-field distribution of the strong-wiggler radiation can be expressed as (Hoffman, 2004 ▸)
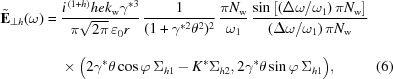
where *h* is the harmonic number, *r* is the observation distance, 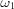 = 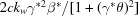 is the fundamental frequency, φ is the azimuthal observation angle, 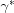 = 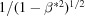, 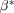 = 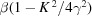 and 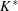 = 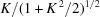. The detailed expressions of 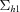 and 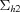 can be written as
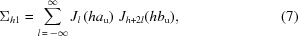


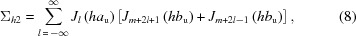
which involve the parameters
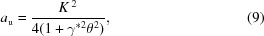


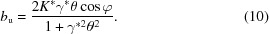
We take the 200 pC THz bunch and 90 cm wiggler period as an example to show the angular distribution of THz radiation. The fundamental resonance frequency is optimized to be at 10.8 THz. In this case, the full spectrum of the radiation including up to the second harmonic is shown in Fig. 17 ▸ with the peak value located at ∼10 THz. Fig. 18 ▸ presents the far-field distribution in the horizontal and vertical angles 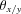 of the THz radiation calculated by equation (6). The lineouts of the energy density at 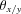 = 0 are also shown in the figure. The total integrated pulse energy over the full bandwidth is 1178 µJ.

**Figure 19:**
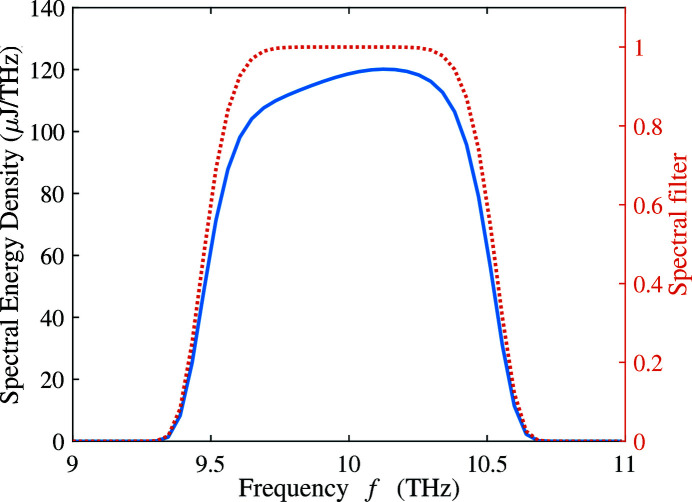
Spectrum of the THz radiation within 10% bandwidth around 10 THz of the 200 pC THz bunch with only the first harmonic. The red dotted line is the shape of the adopted super-Gaussian spectral filter. The resonance frequency on-axis is set at 10.8 THz.

**Figure 20:**
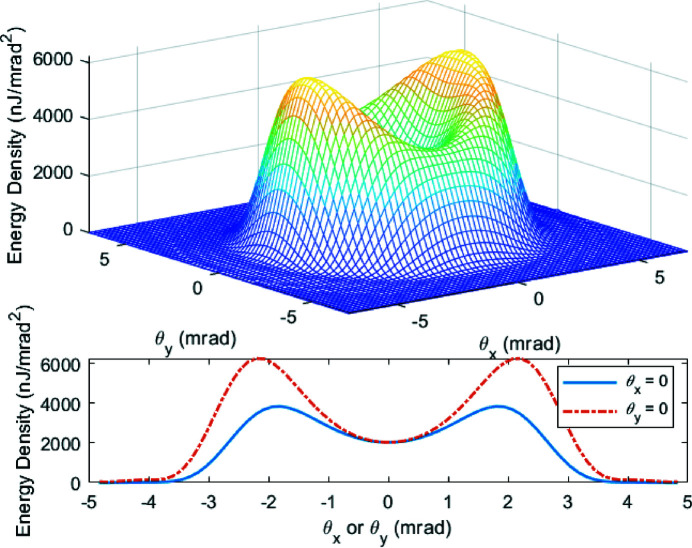
Far-field distribution of the THz radiation calculated by equation (6) over 10% bandwidth around 10 THz of the 200 pC THz bunch with only the first harmonic. The resonance frequency on-axis is set at 10.8 THz.

Applying a 10% bandwidth super-Gaussian filter centered at 10 THz (9.5 THz to 10.5 THz in FWHM), we recalculate the THz radiation with only the first harmonic. The spectrum within the bandwidth is shown in Fig. 19 ▸. The peak value around 10 THz is smaller than the one in Fig. 17 ▸ due to the exclusion of the second harmonic. The corresponding far-field distribution of the energy density is presented in Fig. 20 ▸ with two lineouts at 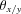 = 0. As the resonance frequency on-axis is 10.8 THz, the main frequency components in the spectrum around 10 THz are off-axis (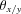 = ±2 mrad). The total integrated pulse energy is 117 µJ, which agrees with the energy estimated in Fig. 16 ▸. The collection angle in the wiggler can be estimated by 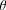 = 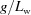 where 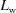 = 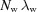 is the total wiggler length. With a 50 mm gap in a ten-period wiggler, 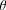 = 5.6 mrad, which is larger than the angular divergence of the 10 THz radiation.

## 6. THz transport   

Soft X-rays follow a nearly straight path from the undulator to the user hutches in the Near Experimental Hall, with only small-angle steering by grazing-incidence mirrors. Optical propagation of the strongly diffracting THz requires large-diameter tubing (200 mm) with frequent reimaging (every 12 to 15 m). The imaging uses reflective optics, due to the wide bandwidth and the lack of good refractive materials. Either off-axis paraboloidal (OAP) or toroidal mirrors may be used, with 45° incidence. Imaging is maintained by separating adjacent mirrors by a distance equal to the sum of their focal lengths. When a 90° change in beam direction is not wanted, the imaging mirror is followed by a flat mirror to redirect the optical path. Since water vapor absorbs THz, the tubing should be evacuated to 1 Pa or below. (Alternatively, the tubing could be filled with nitrogen or another gas that is not excited by THz in this range.)

**Figure 21:**
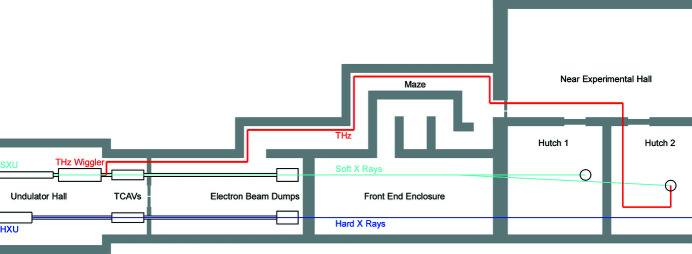
Schematic of THz transport from the end of LCLS undulator hall through the maze to the Near Experimental Hall.

Since the optical path cannot be straight, we can avoid the need to send it through the shielding wall by instead following the turns in the access maze to the beam dump from the Near Experimental Hall (see Fig. 21 ▸). A preliminary layout requires a combination of 12 focusing and 22 flat mirrors to reach one of the principal hutches. Fortunately, in this frequency range reflection losses are below 1% per surface, providing an overall transmission of 70%.

The time between the two electron bunches is determined by the extra path of the THz compared with the straight X-ray path. The THz path through the maze requires an additional 108 ns for simultaneous arrival of the THz pump and the X-ray probe pulses. Pump–probe experiments need a pump that arrives earlier than the probe, with a delay that can be scanned. The THz bunch can be shifted to earlier times by stepping its laser pulse at the gun by an integer number of RF periods. For intervals less than one period (5.4 ns), a motorized ‘optical trombone’ delay line on the wall of the maze adjusts the path by ±1 m. This combination allows an arbitrary separation of the two bunches, limited by the flat-top region of the kicker magnet that directs bunches into the SXR line.

## 7. Summary   

In this paper, we study the two-bunch dynamics in order to generate a highly compressed first bunch for efficient THz generation in a ten-period wiggler following the soft X-ray undulator in LCLS II. A bunch charge of 100–200 pC can be used together with an electromagnet wiggler to produce 3–20 THz with about 100 µJ of pulse energy at a 100 kHz (or higher) repetition rate that is well synchronized with the X-ray pulses. The number of electromagnet wiggler periods can be controlled by power supplies to produce a single-cycle THz pulse with similar pulse energies. When properly focused, the electric field generated by such a THz source will be well above 10 MV cm^−1^. Finally, the wiggler can also be tuned to generate intense infrared and ultraviolet radiation that is well synchronized with the X-ray pulses, and hence significantly enhances pump–probe capabilities at LCLS II.

## Figures and Tables

**Table 1:** Required THz characteristics for both single and ten-cycle pulses for pump–probe experiments at LCLS II

	Single cycle		Ten cycles	
E-field	10 MV cm^−1^		1 MV cm^−1^	
Frequency	5 THz	15 THz	5 THz	15 THz
Spot size	200 µm	100 µm	200 µm	100 µm
Pulse duration	100 fs	33 fs	1000 fs	330 fs

**Table 2:** Parameters of the LCLS II injector

Parameter	Value	Unit
Drive laser		
Beam charge *Q*	100	pC
Laser temporal profile	Flat-top	
Flat-top FWHM 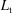	32.91	ps
Flat-top edge width 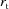	2	ps
Transverse r.m.s. size 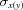	0.1923	mm
Thermal emittance 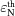	0.1923	µm

VHF gun		
RF frequency of gun 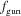	186	MHz
Maximum acceleration gradient 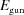	20.04	MV m^−1^
Injection phase 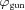	−6.5959†	degree‡

Buncher		
RF frequency of buncher 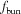	1.3	GHz
Maximum acceleration gradient 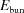	1.7942	MV m^−1^
Acceleration phase 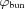	−80.2745	degree
Cavity position 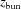	0.8093	m

Eight-cavity cryomodule		
First cavity position 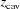	3.3428	m
RF frequency of cavity 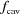	1.3	GHz
Maximum gradient of 1st cavity 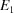 §	15.8	MV m^−1^
Acceleration phase of 1st cavity 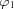	−4	degree
Maximum gradient of 2nd/3rd cavities	0	MV m^−1^
Maximum gradient of 4th cavity	28	MV m^−1^
Acceleration phase of 4th cavity	0	degree
Maximum gradient of 5th–8th cavities	32	MV m^−1^
Acceleration phase of 5th–8th cavities	0/0/1.25/6	degree

† Relative phase to the maximum acceleration.

‡ Degree in the corresponding cavity RF frequency.

§ The gradient refers to the maximum gradient in the cavity and the average gradient can be estimated by the energy gain over the whole cavity.

**Table 3:** Parameters of the LCLS II two-stage acceleration and compression

Parameter	Value	Unit
L1 phase 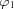	−17.44	degree
L1 energy gain 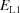 †	181	MeV
L1C phase 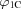	−148.968	degreeC‡
L1C energy gain 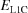	49.9	MeV
BC1 *R*_56_	−52.9	mm
BC1 beam energy 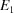	224.36	MeV
L2 phase 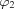	−20.754	degree
L2 energy gain 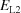	1.4828	GeV
BC2 *R*_56_	−41.1	mm
BC2 beam energy 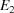	1.6109	GeV
L3 phase 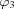	0	degree
L3 energy gain 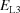	2.3891	GeV

† Energy gain at on-crest acceleration.

‡ Degree in the corresponding cavity RF frequency.

**Table 4:** Effective field, wiggler *K* and radiation frequency *f* at different coil currents for the electromagnet of Fig. 15 ▸

Current (A)	*B*_eff_ (T)	*K*	*f* (THz)
55	0.338	28.4	101
137	0.841	70.7	16.3
219	1.34	112	6.46
301	1.75	147	3.79
383	2.00	168	2.89

## References

[bb1] Bane, K. & Emma, P. (2005). *Proceedings of the 2005 Particle Accelerator Conference*, 16–20 May 2005, Knoxville, TN, USA, pp. 4266–4268.

[bb2] Beukers, T. (2019). Private communication.

[bb3] Beukers, T., Nguyen, M. & Tang, T. (2018). *Proceedings of the 2018 IEEE International Power Modulator and High Voltage Conference (IPMHVC)*, pp. 151–155. IEEE.

[bb4] Chen, F., Zhu, Y., Liu, S., Qi, Y., Hwang, H. Y., Brandt, N. C., Lu, J., Quirin, F., Enquist, H., Zalden, P., Hu, T., Goodfellow, J., Sher, M.-J., Hoffmann, M. C., Zhu, D., Lemke, H., Glownia, J., Chollet, M., Damodaran, A. R., Park, J., Cai, Z., Jung, I. W., Highland, M. J., Walko, D. A., Freeland, J. W., Evans, P. G., Vailionis, A., Larsson, J., Nelson, K. A., Rappe, A. M., Sokolowski-Tinten, K., Martin, L. W., Wen, H. & Lindenberg, A. M. (2016). *Phys. Rev. B*, **94**, 180104.

[bb5] Decker, F.-J., Van Hoover, Z., Loos, H., Marinelli, A., Stan, C., Huang, Z., Turner, J., Vetter, S. & Gilevich, S. (2015). *Proceedings of the 37th International Free Electron Laser Conference (FEL 2015)*, 23–28 August 2015, Daejeon, Korea, p. 725.

[bb6] Floettmann, K. (2017). *ASTRA – Space Charge Tracking Algorithm.* Technical Report. DESY, Hamburg, Germany.

[bb7] Gaffney, K., Goldstein, J., Kirchmann, P., Lindenberg, A. & Shen, Z.-X. (2012). *Frontier of THz Science.* Technical Report. SLAC National Accelerator Laboratory, Menlo Park, CA, USA.

[bb8] Geloni, G., Tanikawa, T. & Tomin, S. (2019). *J. Synchrotron Rad.* **26**, 737–749. 10.1107/S1600577519002509 31074438

[bb9] Gray, A. X., Hoffmann, M. C., Jeong, J., Aetukuri, N. P., Zhu, D., Hwang, H. Y., Brandt, N. C., Wen, H., Sternbach, A. J., Bonetti, S., Reid, A. H., Kukreja, R., Graves, C., Wang, T., Granitzka, P., Chen, Z., Higley, D. J., Chase, T., Jal, E., Abreu, E., Liu, M. K., Weng, T.-C., Sokaras, D., Nordlund, D., Chollet, M., Alonso-Mori, R., Lemke, H., Glownia, J. M., Trigo, M., Zhu, Y., Ohldag, H., Freeland, J. W., Samant, M. G., Berakdar, J., Averitt, R. D., Nelson, K. A., Parkin, S. S. P. & Dürr, H. A. (2018). *Phys. Rev. B*, **98**, 045104.

[bb10] Green, B., Kovalev, S., Asgekar, V., Geloni, G., Lehnert, U., Golz, T., Kuntzsch, M., Bauer, C., Hauser, J., Voigtlaender, J., Wustmann, B., Koesterke, I., Schwarz, M., Freitag, M., Arnold, A., Teichert, J., Justus, M., Seidel, W., Ilgner, C., Awari, N., Nicoletti, D., Kaiser, S., Laplace, Y., Rajasekaran, S., Zhang, L., Winnerl, S., Schneider, H., Schay, G., Lorincz, I., Rauscher, A. A., Radu, I., Mährlein, S., Kim, T. H., Lee, J. S., Kampfrath, T., Wall, S., Heberle, J., Malnasi-Csizmadia, A., Steiger, A., Müller, A. S., Helm, M., Schramm, U., Cowan, T., Michel, P., Cavalleri, A., Fisher, A. S., Stojanovic, N. & Gensch, M. (2016). *Sci. Rep.* **6**, 22256. 10.1038/srep22256 PMC4770290 26924651

[bb11] Halbach, K. (1981). *Nucl. Instrum. Methods Phys. Res.* **187**, 109–117.

[bb12] Hauri, C. P., Ruchert, C., Vicario, C. & Ardana, F. (2011). *Appl. Phys. Lett.* **99**, 161116.

[bb13] Hebling, J., Almasi, G., Kozma, I. & Kuhl, J. (2002). *Opt. Express*, **10**, 1161–1166. 10.1364/oe.10.001161 19451975

[bb15] Hoffmann, M. C. & Fülöp, J. A. (2011). *J. Phys. D Appl. Phys.* **44**, 083001.

[bb14] Hoffmann, M. C., Grguraš, I., Behrens, C., Bostedt, C., Bozek, J., Bromberger, H., Coffee, R., Costello, J. T., DiMauro, L. F., Ding, Y., Doumy, G., Helml, W., Ilchen, M., Kienberger, R., Lee, S., Maier, A. R., Mazza, T., Meyer, M., Messerschmidt, M., Schorb, S., Schweinberger, W., Zhang, K. & Cavalieri, A. L. (2018). *New J. Phys.* **20**, 033008.

[bb16] Hofmann, A. (2004). *The Physics of Synchrotron Radiation*, Vol. 20. Cambridge University Press.

[bb17] Kim, K.-J., Huang, Z. & Lindberg, R. (2017). *Synchrotron Radiation and Free-Electron Lasers.* Cambridge University Press.

[bb18] Kozina, M., Fechner, M., Marsik, P., van Driel, T., Glownia, J. M., Bernhard, C., Radovic, M., Zhu, D., Bonetti, S., Staub, U. & Hoffmann, M. C. (2019). *Nat. Phys.* **15**, 387–392.

[bb19] Kubacka, T., Johnson, J. A., Hoffmann, M. C., Vicario, C., de Jong, S., Beaud, P., Grubel, S., Huang, S. W., Huber, L., Patthey, L., Chuang, Y. D., Turner, J. J., Dakovski, G. L., Lee, W. S., Minitti, M. P., Schlotter, W., Moore, R. G., Hauri, C. P., Koohpayeh, S. M., Scagnoli, V., Ingold, G., Johnson, S. L. & Staub, U. (2014). *Science*, **343**, 1333–1336. 10.1126/science.1242862 24603154

[bb20] MacArthur, J. P., Duris, J., Zhang, Z., Lutman, A., Zholents, A., Huang, Z. & Marinelli, A. (2019). *Phys. Rev. Lett.* **123**, 214891. 10.1103/PhysRevLett.123.214801 31809147

[bb21] Mecseki, K., Windeler, M. K. R., Miahnahri, A., Robinson, J. S., Fraser, J. M., Fry, A. R. & Tavella, F. (2019). *Opt. Lett.* **44**, 1257–1260. 10.1364/OL.44.001257 30821762

[bb22] Ofori-Okai, B. K., Sivarajah, P., Ronny Huang, W. & Nelson, K. A. (2016). *Opt. Express*, **24**, 5057–5068. 10.1364/OE.24.005057 29092334

[bb23] Penco, G., Allaria, E., Bassanese, S., Cinquegrana, P., Cleva, S., Danailov, M. B., Demidovich, A., Ferianis, M., Gaio, G., Giannessi, L., Masciovecchio, C., Predonzani, M., Rossi, F., Roussel, E., Spampinati, S. & Trovò, M. (2018). *New J. Phys.* **20**, 053047.

[bb24] Perucchi, A., Di Mitri, S., Penco, G., Allaria, E. & Lupi, S. (2013). *Rev. Sci. Instrum.* **84**, 022702. 10.1063/1.4790428 23464184

[bb25] Saldin, E. L., Schneidmiller, E. A. & Yurkov, M. V. (2005). *Nucl. Instrum. Methods Phys. Res. A*, **539**, 499–526.

[bb26] Schmerge, J., Adolphsen, C., Corbett, J., Dolgashev, V., Durr, H., Fazio, M., Fisher, A., Frisch, J., Gaffney, K., Guehr, M. *et al.* (2015). *A Tunable, Linac-based, Intense, Broadband THz Source for Pump–Probe Experiments.* Technical Report SLAC-R-1049. SLAC National Accelerator Laboratory, Menlo Park, CA, USA.

[bb27] Schmerge, J., Brachmann, A., Dowell, D., Fry, A., Li, R., Li, Z., Raubenheimer, T., Vecchione, T. & Zhou, F. (2014). *Proceedings of the 36th International Free-Electron Laser Conference (FEL2014)*, 25–29 August 2014, Basel, Switzerland.

[bb28] Tanikawa, T., Karabekyan, S., Kovalev, S., Casalbuoni, S., Asgekar, V., Serkez, S., Geloni, G. & Gensch, M. (2019). *J. Instrum.* **14**, P05024. 10.1107/S1600577520004245 PMC7206546 32381783

[bb29] Tibai, Z., Tóth, G., Mechler, M. I., Fülöp, J. A., Almási, G. & Hebling, J. (2014). *Phys. Rev. Lett.* **113**, 104801. 10.1103/PhysRevLett.113.104801 25238363

[bb30] Tiedtke, K., Azima, A., von Bargen, N., Bittner, L., Bonfigt, S., Düsterer, S., Faatz, B., Frühling, U., Gensch, M., Gerth, C., Guerassimova, N., Hahn, U., Hans, T., Hesse, M., Honkavaar, K., Jastrow, U., Juranic, P., Kapitzki, S., Keitel, B., Kracht, T., Kuhlmann, M., Li, W. B., Martins, M., Núñez, T., Plönjes, E., Redlin, H., Saldin, E. L., Schneidmiller, E. A., Schneider, J. R., Schreiber, S., Stojanovic, N., Tavella, F., Toleikis, S., Treusch, R., Weigelt, H., Wellhöfer, M., Wabnitz, H., Yurkov, M. V. & Feldhaus, J. (2009). *New J. Phys.* **11**, 023029.

[bb31] Wen, H., Kim, K., Zholents, A., Byrd, J. & Cavalleri, A. (2013). *Rev. Sci. Instrum.* **84**, 022501. 10.1063/1.4790426 23464182

[bb32] Windeler, M. K. R., Mecseki, K., Miahnahri, A., Robinson, J. S., Fraser, J. M., Fry, A. R. & Tavella, F. (2019). *Opt. Lett.* **44**, 4287–4290. 10.1364/OL.44.004287 31465384

[bb33] Wu, Z., Fisher, A. S., Goodfellow, J., Fuchs, M., Daranciang, D., Hogan, M., Loos, H. & Lindenberg, A. (2013). *Rev. Sci. Instrum.* **84**, 022701. 10.1063/1.4790427 23464183

[bb34] Zalden, P., Carley, R., Tschentscher, T., Bressler, C., Molodtsov, S., Geloni, G., Scherz, A. *et al.* (2018). *Terahertz Science at European XFEL*, Technical Note XFEL.EU TN-2018-001-01.0. EuropeanX-ray Free-Electron Laser Facility GmbH, Schenefeld, Germany.

[bb35] Zhang, X., Ma, X. F., Jin, Y., Lu, T., Boden, E. P., Phelps, P. D., Stewart, K. R. & Yakymyshyn, C. P. (1992). *Appl. Phys. Lett.* **61**, 3080–3082.

